# Interleukin-8 in Melanoma Pathogenesis, Prognosis and Therapy—An Integrated View into Other Neoplasms and Chemokine Networks

**DOI:** 10.3390/cells11010120

**Published:** 2021-12-30

**Authors:** Anca Filimon, Iulia A. Preda, Adina F. Boloca, Gabriela Negroiu

**Affiliations:** Group of Molecular Cell Biology, Institute of Biochemistry of the Romanian Academy, 060031 Bucharest, Romania; afilimon@biochim.ro (A.F.); iuliapreda30@gmail.com (I.A.P.); adina.fdobre@gmail.com (A.F.B.)

**Keywords:** interleukin-8, chemokines, CXCL8, CXCR1, CXCR2, chemokines in tumor progression

## Abstract

Cutaneous melanoma accounts for only about 7% of skin cancers but is causing almost 90% of deaths. Melanoma cells have a distinct repertoire of mutations from other cancers, a high plasticity and degree of mimicry toward vascular phenotype, stemness markers, versatility in evading and suppress host immune control. They exert a significant influence on immune, endothelial and various stromal cells which form tumor microenvironment. The metastatic stage, the leading cause of mortality in this neoplasm, is the outcome of a complex, still poorly understood, cross-talk between tumor and other cell phenotypes. There is accumulating evidence that Interleukin-8 (IL-8) is emblematic for advanced melanomas. This work aimed to present an updated status of IL-8 in melanoma tumor cellular complexity, through a comprehensive analysis including data from other chemokines and neoplasms. The multiple processes and mechanisms surveyed here demonstrate that IL-8 operates following orchestrated programs within signaling webs in melanoma, stromal and vascular cells. Importantly, the yet unknown molecularity regulating IL-8 impact on cells of the immune system could be exploited to overturn tumor fate. The molecular and cellular targets of IL-8 should be brought into the attention of even more intense scientific exploration and valorization in the therapeutical management of melanoma.

## 1. Introduction

The pathogenesis of each type of neoplasm is defined by individual molecular mechanisms acting specifically in different stages. However, despite these particularities, there is a general common scenario according to which various types of cancers, including cutaneous melanoma (CM), evolve.A normal cell acquires mutations and does not follow the contact-inhibition rule anymore, thereby allowing uncontrolled growth. The “no turning back point” in the evolution of most tumors is the dissemination from primary site. The transformed cells switch to an invasive phenotype, cross the basement membranes, enter the circulatory systems and spread to distal organs where they start to grow again. Numerous of these complex processes are regulated by molecular mediators, referred generally as cytokines (CyKs), which include *chemokines*, interferons, interleukins, lymphokines and tumor necrosis factors. In cancer progression, the CyKs act in conjunction with other classes of molecules in cell-contact mechanisms and tumor intrinsic events.

**Figure 1:**
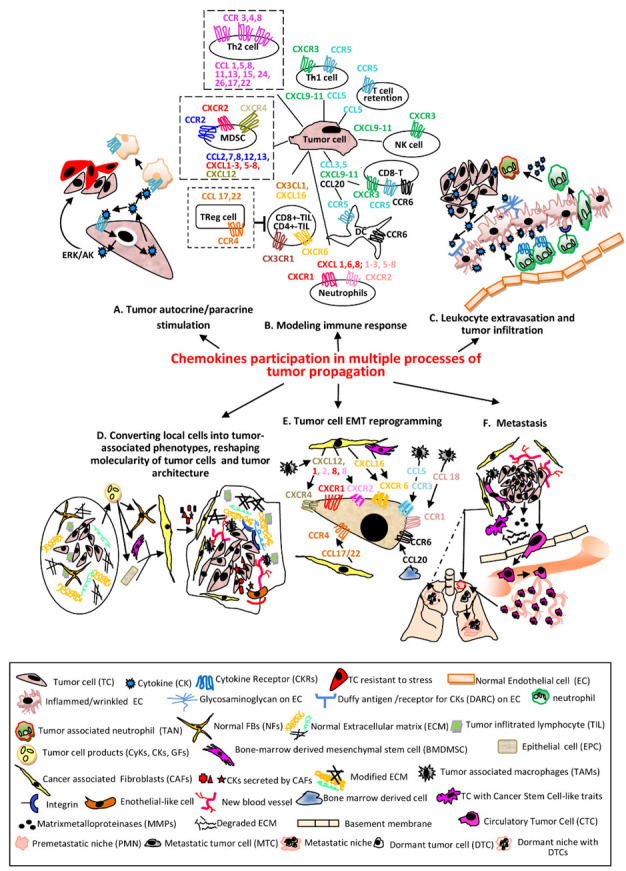
The main processes regulated in various cellular phenotypes by CKs in tandem with their receptors. (**A**) TCssynthetize and secrete CKs which bind to self CKRs or CKRs expressed by other cells resulting activation of signaling pathways which sustain proliferation, survival and stress-resistance of the same TC (autocrine) or adjacent phenotypes (paracrine) [5]. (**B**) The CKs secreted by TCs recruit different immune cells in tumor micro environment (TME). The most relevant and documented examples of CKs/CKRs axes are presented. The pro-tumoral associated with poor prognosis are shown with dotted lines (NK—natural killer; Th1/Th2-CD4^+^T lymphocytes helper type1/type2; CD8^+^/CD4^+^-TIL-CD4^+^/CD8^+^ Tumor infiltrating lymphocyte; Treg-Regulatory T cell; MDSC—myeloid derived suppressor cell; DC—dendritic cell) [6,7]. (**C**) CKs secreted by TCs bound to the receptors (glycosaminoglycans and Duffy antigen) of inflamed (wrinkled) ECs are transcytosed and accumulated onto luminal face of ECs; the leucocytes migrate into lumen of the vessel toward CKs and establish firm attachments via overexpressed integrins, traverse endothelium through a gap junction or a pore, follow the extracellular CK gradient and enter the TME becoming TANs [8]. (**D**) Primary tumors contain NFBs and ECM with an ordered composition adequate to ensure immune infiltrates (left). Secreted factors by TCs convert NFBs and other Stromal Cells (SCs) into CAFs or Tumor Stromal Cells (TSCs-BMDMSCs or EPCs); here for simplicity only CAFs are presented. TSCs release various CKs which increase the number of stationary TAMs, that secrete CKs pro-tumor cell survival, induce angiogenesis, sustain the proliferative, migratory and invasive processes in TCs and vascular mimicry phenomenon, represented by Endothelial-like cells. The advanced tumor ECM composition becomes stiffer, includes modified proteins, stronger interactions via overexpressed integrins, keeping chemotherapeutic drugs and immune cells at tumor periphery (right) [9,10,11]. (**E**) The process of endothelial to mesenchymal transition (EMT) is essential in enabling TCs for invasion and metastatic dissemination. The most significant CKs secreted by TSCs acting on CKRs expressed on TCs and involved in EMT are presented [12]. (**F**) The cell signaling pathways activated by CKs in TCs and TSCs define the metastasizing process with the following major events: *Inducing Cancer Stem Cell-like traits* in TCs (self-renewal, colonies formation, mesenchymal multipotent differentiation) [13]. *Remodeling ECM and invasion*—the increased MMPs in TME disassemble components of ECM; TCs acquire invadopodia, protrude into the basement membranes, advance and disseminate within surrounding tissues [14]. *Intravasation*—TCs migrate across the vessel into blood or lymph circulatory systems and become CTCs [15]. *Death or survival within circulatory system*—until the opportunity of extravasation occurs, CTCs have to overcome hurdles as: lack of GFs/CytKs, the circulatory flow, anoikis, anti-tumor mechanisms of activated NKs/phagocytic cells and form circulating tumor microemboli [16]. *Pre-metastatic niches (PMNs) formation*—in secondary organs, as result of migration of hematopoietic bone marrow cells and SCs to particular sites in distal organs, with remodeled ECM, occur environmental milieus favorable to later installation and proliferation of TCs. These events facilitate the colonization with TCs, CyKs, GFs and TCs-derived exosomes as well as the suppression of immune system and installation of hypoxia. Distinct PMNs are formed in different organs [17,18]. In addition, dormant niches (DNs) (dotted line) with opposite properties from PMNs are also formed. *Extravasation*—TCs leave circulatory system and are seeded in PMNs [19]. *Dissemination*—Unlike DNs which will promote tumor cell dormancy, PMNs will sustain metastatic outgrowth instead. A primary tumor has the ability to seed more than one organ. PMNs are formed in different organs, which control the fate of TCs seeding at each metastatic site. A tumor contains sub-clones which colonize specific organs forming organ-specific metastasis [18].

Chemokines (CKs), also known as chemotactic cytokines (CyKs) (Greek kinos-movement), are small polypeptides of 8-10 kDa, capable to induce chemotaxis in their neighboring cells [1]. There are 48 CKs (referred to also as ligands-L/CKLs) grouped in four major subclasses according to structural particularities, XCL, CCL, CXCL and CX3CL. Their receptors (CKRs) named accordingly are associated with G-proteins and bind several ligands from the same family. The CKs in tandem with their CKRs are involved in tumor pathogenesis through numerous complex processes [2,3,4].Every step of the way they engage different cell programs to fulfill required tasks in both tumor rejection and favor as well. The participation of CKs/CKRs in various processes and the molecularity behind each indicated event are schematically represented and commented in Figure 1. The secreted CKs induce autocrine and paracrine stimulation in self tumor cells or in other cell types, respectively (Figure 1A), have an impact on both pro-or anti-tumor immune responses (Figure 1B), mediate leukocyte tumor infiltration (Figure 1C), reshape TME by reprogramming different cell phenotypes mainly in tumor favour (Figure 1D,E) and are key players in all metastatic progression events (Figure 1F).

The name of Interleukin-8 (IL-8) is emblematic for the “birth of chemokine field” [20]. IL-8 is contributive to normal and temporary pathological processes. IL-8 induces the attraction of specific leukocyte populations during the peri-implantation phase of the embryo [21]. In infections and injuries, IL-8, released by macrophages, epithelial or endothelial cells, induces attraction of neutrophils to the affected sites. In response to this event, the clearance of pathogens, the activation of angiogenic response, and the formation of new blood vessels occur [22,23].

The role of IL-8 is continuously expanding. From the intensely acknowledged pro-inflammatory chemokine investigated mainly in chronic inflammatory diseases [24] and target for possible anti-inflammatory therapeutic strategies [25,26] to that of main tumor regulator [27,28,29,30] and most recently, biomarker for SARS-COV2 viral infections [31]. The impact of IL-8 and its receptors on melanoma growth and metastasis has been evaluated in several previous reviews [32,33,34].

Our present work firstly aimed to update the status of IL-8 in melanoma cellular complexity. We also considered that describing comparatively IL-8 participation in other neoplasms and in relation with other CKs would be important for a more comprehensive understanding of common versus specific melanoma mechanisms. Presently, IL-8 is by far more than just a chemoattractant for cells of the immune system; it is a multi-task molecular modulator of numerous cellular phenotypes which build melanoma tumor tissue. The accumulating evidence of IL-8 activities in melanoma, stromal and vascular cells and cells of the immune system unveiled orchestrated mechanisms within cytokine signaling webs operating in melanoma pathogenesis. The significance of IL-8 produced in large quantities by melanoma cells and IL-8-mediated pathways, which have just emerged to be exploited in melanoma prognostic and therapies, are comparatively reviewed with the IL-8-operating ones in other neoplasms.

## 2. Melanoma Progression—An Interplay between Chemokines, Chemokine Receptors and Cellular Phenotypes within Tumor Microenvironment

Cutaneous melanoma (CM) accounts for only about 7% of skin cancers but is causing almost 90% of deaths. The prevalence of CM is continuously increasing. A study about the incidence rate and new cases estimates that in US the number of patients will rise to 116,000 in 2026–2031 [35]. CM is mainly due to DNA damage in response to UV light of melanocytes, the cells that normally reside at basal dermal-epidermal layer and are responsible of melanin pigment production. The most agreed scenario for CM development is the step-wise one [36]. CM is initiated when melanocytes accumulate somatic mutations which affect genes involved in proliferation (BRAF, NRAS), growth and metabolism (phosphatase and PTEN), proto-oncogene receptor tyrosine kinase (KIT), resistance to apoptosis and cell cycle (p53, CDKNA2) and replicative lifespan (TERT) [37,38]. They escape from keratinocyte control and become highly proliferative forming benign tumors (nevi). In some individuals the phenotype switch continues with dysplastic nevus, the precursor of the malignant lesions. In a more advanced stage, the cells in radial growth phase (RGP) are proliferative but limited to epidermis and have low invasive potential. In the vertical growth phase (VGP) cell populations migrate vertically up into the epidermis and down into the papillary dermis. In metastatic stage, the tumor cells invade through blood or lymph vessels into distal organs (liver, brain and lung) where they proliferate, eventually, causing death.

The metastatic stage, which remains by far the leading cause of deaths in CM, is the outcome of a complex, still poorly understood, cross-talk between tumor and other cell phenotypes. Melanoma cells (MCs) have the following unique characteristics: a repertoire of mutations distinct from other cancers, surface molecules similar to vascular cells, a high plasticity and degree of mimicry toward vascular phenotype, stemness markers, communication via exosomal vesicles, and versatility in evading and suppressing host immune control [39]. Overall, MCs exert a significant influence on immune, endothelial and various stromal cells which form melanoma/tumor microenvironment (MME/TME) [40].

One common characteristic of all these cell phenotypes is the expression of CKs, which in conjunction with other CyKs orchestrate the dynamics of melanoma tumor, progression or rejection [41,42,43]. The impact of CKs resides in their expression together with corresponding CKRs, which act, on one hand, on melanoma or different tumor stromal cells, and on the other hand, on immune cells. The first set ensure tumor cell auto-stimulation, reprogramming, metastatic dissemination whereas the second set controls the tumor fate. In correlation with these issues, several CKs-CKRs axes have been documented based on data obtained with the following: human melanoma cell lines, murine melanoma models, fresh or archived tissue specimens, and studies with specific antibodies versus CKs or CKRs [44]. The meta-analysis of such axes can anticipate metastatic sites and tumor fate from correlation with specific CKRs on the surface of melanoma and immune cells, respectively. For example, melanoma cells expressing CCR9 are often expected to metastasize in the intestine [45], CCR6 or CXCR4 in lung [46] and liver [47], CCR4 associated with brain metastases [48], whereas cells with high expression of CCR10, CCR7, and CXCR3 populate regional lymph nodes [49]. The CCR10 expressed on CD4^+^ T effector memory cells are associated with worse survival [50], whereas CXCR3 on blood and CD4^+^/CD8^+^ T-effector memory cells, critical in intra-tumoral T cell trafficking, are associated with clinical benefits [51,52]. The combinatorial analysis of CKs/CKRs on melanoma and immune cells results in obtaining the molecular anatomy of melanoma stage, the tissular localization and possibly more efficient therapeutic targeting [43,44]. Several therapeutic approaches are currently taken into account, as follows: blocking CKRs with specific antibodies [53,54], bispecific antibodies constructed to pass the blood–brain barrier by binding both CKRs and brain endothelial cell receptors [55], induction of tertiary lymphoid structures in the so-called “cold” tumors positively associated with immune cell activation and patient survival [56].

## 3. Interleukin 8—A Major Key-Player in CM Pathogenesis

Human interleukin-8 (IL-8) has been renamed as chemokine (C-X-C motif) ligand 8 and its approved gene symbol is *CXCL8* and its receptors alpha and beta are CXCR1 and CXCR2, respectively. In the present material this cytokine will be referred as IL-8 and its receptors as IL-8Rs, for simplicity. IL-8 was the first cytokine-like chemoattractant with cell specificity, discovered in the culture supernatant of Lipopolysaccharide (LPS)-activated peripheral blood mononuclear cells (PBMCs), originally called “human monocyte-derived neutrophil chemotactic factor” [57].

### 3.1. The Cellular Expression of IL-8 and IL-8Rs

IL-8 functions in tandem with G-protein coupled receptors, CXCR1 and CXCR2 which belong to the G-protein coupled receptor (GPCR) family, have 7 transmembrane domains and form a gene cluster on chromosome 2q33-q36 [58]. IL-8 binding to IL-8Rs induces conformational changes in receptor intracellular loops and C-tail and couple them to heteromeric small G-proteins (Gαβγ). The multiple cell types which express IL-8 and IL-8Rs presented in Table 1 [23,28,30,59,60,61,62,63,64,65,66,67,68,69,70,71,72,73,74,75,76,77,78,79,80,81,82,83,84,85]. This diversity demonstrates how the fate of other tissues may interfere with the evolution of the neoplastic cells, including melanoma, which express both IL-8 and IL-8Rs. CXCR1 is activated only in response to IL-8 and CXCL6 whereas CXCR2 is activated by multiple CXCLs (1–3, 5–8). CXCR2 is known to mediate angiogenetic activity of ELR^+^ CKs upon their binding. Importantly, the ligand binding to CXCR2 does not trigger only the downstream activatory signals but may also induce receptor-mediated endocytosis, via clathrin-coated vesicles, which practically abort CXCR2 availability [86].

The distribution of CXCR1/2 is different in various cell types. For example, CXCR2 is better expressed in melanoma than in neutrophils [1], which automatically increases IL-8 binding to tumor instead to immune cells with all the subsequent events which may arise from this. The CXCLs that commonly bind to IL-8Rs are named CXCL8 family [87]. Recently two new ligands of CCR1/CXCR2, namely PGP (a peptide resulted from ECM degradation) [88] and MIF (cytokine macrophage migration inhibitory factor) [89] have been discovered. The expression variability of CXCR1/2 together with the availability of multiple ligands, dynamically regulated by TME, demonstrate that the complex processes in which IL-8 axis activates are far from being elucidated.

### 3.2. Molecular Regulators of IL-8 Expression in CM

The transcription of IL-8 gene responds to pro-inflammatory mediators and its regulators are highly ordered and cell-specific [90]. IL-8 is a downstream target of nuclear factor of activated T cell (NFAT1, NFATC2), a transcription factor with important roles in adaptive and innate immune responses as well as in melanoma progression and metastasis. NFAT1 positively regulates the expression of IL-8 through the binding to IL-8 promoter. This event enhances IL-8 transcription thus contributing to melanoma growth and metastasis [91]. IL-8 expression is also regulated by constitutive nuclear factor kappa-light-chain-enhancer of activated B cells (NF-κB). This is a protein complex which controls DNA transcription, cell survival, production of CKs and immune responses to infections as well. NF-kB is thus a well-recognized link between immunity and tumorigenesis. In melanoma mice models, NF-kB activity is well-correlated with tumor growth, neovascularization and metastasis. NF-κB is essential for IL-8 expression since mutations in its binding site completely abrogate the IL-8 promoter activity [92].

**Table 1:** The expression of IL-8 and IL-8Rs in different cell phenotypes.

Cell Type	IL-8/References	Cell Type	IL-8Rs/References
Tumor cells	[28,30,59]	Tumor cells	[30,59]
Melanocytes/melanoma	[60]	Melanocytes/melanoma	[60]
Tumor stem cells	[71,79]	Keratinocytes	[63,84]
Endothelial cells	[23]	Neurons and glial cells	[64]
Epithelial cells	[80]	Hepathocytes	[65]
Fibroblasts	[81]	Endothelial cells	[66]
Cancer associated Fibroblasts	[82]	Epithelial cells	[67]
Keratinocytes	[83,84]	Neutrophils	[30,68,74]
Synovial cells	[61]	CD8+ T cells	[68,75]
Smooth muscle cells	[62]	Mast cells	[68,75]
Monocytes	[69,70]	Natural killer	[68,76]
Macrophages	[72,85]	Myeloid derived suppressor cell	[68,77]
T-cell lymphocytes	[73]		
Regulatory T cell	[78]		

The role of NF-κB signaling in IL-8 expression is underlined by the effect of miR-7-5p on the RelA (p65) subunit of NF-κB and the downstream target genes. In WM266-4, 1205Lu and A2058 melanoma cell lines miR-7-5p targets two sites situated in the 3’ UTR of RelA and is inversely correlated with mRNA and protein levels. miR-7-5p inhibits NF-κB transcriptional activity and reduces secretion and intracellular expression of IL-1β, IL-6 and IL-8 [93]. NF-κB is also regulated by FKBP51 (FK506-binding protein 51), a member of the immunophilin family that has peptidyl-prolyl isomerase activity and is able to bind the immunosuppressants rapamycin and FK506 [94]. The drug efflux transporter ABCB5 (ATP-binding cassette, sub-family B (MDR/TAP member 5) is another controller of IL-8 expression in melanoma cells through a mechanism that involves whey acidic protein 4-disulphide core domain 1 (WFDC1/ps20) and Wnt pathway [95]. ABCB5 knockdown in G3361 and A375 cell lines induces upregulation of tumor suppressor WFDC1, downregulation of several Wnt ligands and of IL-8 at both mRNA and protein levels. The IL-8 promotor contains also a repressor element, Oct 1 [96] and its activity is downregulated by HPV-E6 protein which competes with NF-κB/p65 and SRC-1 for binding to the N terminus and C terminus of the coactivator CBP, respectively [97]. The signaling via CXCR3 also results in a significant increase inIL-8 expression in BRAF wild type melanomas and these two events have been also associated with melanoma progression [98]. Post-transcriptionally, IL-8 expression is regulated byTip 110/SART3 (HIV-1 Tat-interacting protein), a nuclear protein that impacts IL-8 mRNA stability. Actinomycin D experiments revealed that Tip 110 knockdown prolongs IL-8 mRNA half-life by a p38 MAPK dependent mechanism. Treatment of 1205Lu melanoma cells with SB203580 p38 MAPK signaling inhibitor led to a decrease in IL-8 up-regulation at the mRNA level in cells where Tip110 was downregulated through small interfering RNA [99].

### 3.3. IL-8 (CXCL8)/IL-8Rs(CXCR1,CXCR2) Axis in CM Progression

We addressed here the IL-8/IL-8Rs axis taking into consideration the dynamic relations between tumor cells and other immune and non-immune cellular phenotypes, which populate MME, as these are all active participants in melanoma formation and consolidation.

#### 3.3.1. Tumor Cells and Immune Cells

These cells establish numerous cross-talks at primary sites, which results in switching the molecular repertoire toward a migratory and invasive one. In more advanced stages, tumor cells induce various tumor-reactive cytotoxic mechanisms, or exert immunosuppressive functions to abort the immune attack and ensure tumor escape [100].

##### IL-8 and Melanoma Cells

In melanocytes, IL-8 production has been reported so far only after stimulation with CyKs, like TNF or IL-1β which create premises of pro-inflammatory mechanisms activation [101]. In melanoma cell lines the expression of steady state levels of IL-8 mRNA correlated with their metastatic capacity and no IL-8 mRNA was observed in non-tumorigenic, non-metastatic ones [102]. In malignant lesions, IL-8 expression was negative in RGP, weak to medium in VGP, and highly increased in metastatic specimens. IL-8 became overexpressed upon UV exposure in low-tumorigenic non-metastatic CM and this event significantly increased the tumorigenicity and metastatic potential in nude mice [103]. This is supportive evidence for the role which pro-inflammatory cytokines may have in the pathogenesis of secondary tumor growth (metastasis) at sites of inflammation. Melanoma stem cells, also known as malignant melanoma-initiating cells (MMICs), form a particular niche with strong implications in tumor progression, drug resistance and metastasis [104]. MMICs have self-renewal and differentiation abilities in the context of the continuous exposure to factors from MME, one being the network of pro-inflammatory CKs [105]. There is a cross-talk between IL-8 and ABCB5, which functions as both drug-efflux pump and marker of MMICs, and is associated with clinical tumor progression, therapeutic resistance and recurrence [95]. Due to the plasticity of MMICs and the involvement of IL-8/IL-8Rs in EMT phenotype switching, or in response to hypoxic or toxic milieus, it is rational to assume that this axis is very likely regulating the fate of other markers expressed by MMICs, in stem niche.

In conclusion, melanoma cells expressing IL-8 and other members of IL-8 family function in an autocrine fashion, activating self-oncogenic signaling and pro-metastatic traits like invasion, drug-resistance or angiogenesis.

##### IL-8 and Immune Cells

ME is immunogenic. The natural anti-tumor response includes cells, as tumor-infiltrating lymphocytes (TILs), which are fighters against cancer cells and associated with a positive outcome in CM [106]. Unfortunately, the versatile MCs and MME are not only able to bypass, but also to suppress the immune attack and even to maintain a prolonged, hostile immune milieu, creating one of the most intense therapeutic resistances. The stroma cells are main contributors of tumor immune escape, inducing a rapid recruitment, expansion and activation of various immunosuppressive cells. IL-8/IL-8Rs axis is involved in alterations of the composition of immune infiltrates, resulting the accumulation and activation of such immunosuppressive and pro-tumorigenic immune cells, including the following: *TANs, TAMs, MDSCs, DCs, Tregs* [107]. *IL-8 and TANs*—eutrophils, involved in the first line of immune defense, normally infiltrate into a site of microbial inflammation in order to clear it and in primary tumors as well. MCs by themselves cannot effectively adhere to and migrate through the endothelium under flow conditions. After intravasation, TCs encounter leukocytes which promote their docking along vascular endothelium. IL-1β and IL-6 participate in inducing IL-8 expression in polymorphonuclear leukocytes (PMNs), which in turn enable the extravasation of MCs [108].TANs are neutrophils engaged at tumor site and can be anti-or pro-tumor phenotypes, N1-TANs and N2-TANs, respectively [109]. Tumor-derived IL-8 and related ELR^+^ CKs are key players in N1-/N2-TANs balance by actively suppressing antitumor immunity through the recruitment of N2-TANs. In their turn, TANs can produce IL-8 and other ELR^+^CKs, thereby further augmenting neutrophil migration, angiogenesis and tumor growth. IL-8 induces neutrophil migration, favors the formation of Neutrophils Extracellular Traps (NETs) which trigger a complex apoptotic process called NETosis. In melanoma, ulceration and neutrophil infiltration were previously associated with worse prognosis, but there is a report in which these events were a condition of NETosis associated with tumor necrosis and inhibition of MC migration [110]. The role of IL-8/IL-8Rs axis and NETs in melanoma progression or rejection is presently still a controversial debate. *IL-8 and TAMs*—as neutrophils, macrophages (MPGs) are also in the first line of innate immunity. The blood monocytes, which are the circulating form of MPGs, move into tissues and undergo further differentiation to become multifunctional tissue MPGs. The MCs and TAMs are interacting via soluble factors that either prevent or enhance tumor growth suggesting opposing effects for these soluble factors in CM [111]. The TAMs in CM have dual opposite behavior, as a “hinge” dictated by MME composition. They are either pro-tumor by sustaining angiogenesis, vascularization, stroma formation, dissolution, and modulation of tumor cell growth, or, on the contrary, behave as eradicators of neoplasms acting as phagocytic cells, antigen-presenting cells, activators of T-lymphocytes. TAMs may represent key effectors in tumor angiogenesis and tumor invasivity via IL-8-mediated processes. IL-8 secreted by TAMs demonstrated their involvement in macrophage-derived angiogenesis [112] whereas TAMs secreting IL-6 and IL-8 facilitated the metastasis of colorectal cancer induced by PRL-3 (Phosphatase of regenerating liver) marker [113]. *IL-8 and MDSCs*—DSCs represent an extremely heterogeneous cell population of immature myeloid cells which derive from the bone marrow hematopoietic precursors due to the alteration of myelopoiesis in pathological states as cancer or inflammation. They are one of the most important components of tumor-induced immunosuppression, easily recruited and activated by different inflammatory mediators. MDSCs suppress anti-tumor immune responses by inhibiting T cells via multiple mechanisms [114] as perturbing of T-cell homing, production of the immunosuppressive factors and induction of Tregs [115]. IL-8 recruits into MME highly immunosuppressive MDSCs potentially expanded by IL-6 produced also by melanoma cells. Melanoma patients with high frequencies of MDSCs have decreased overall survival and increased risk of death [116]. *IL-8 and DCs*—DCs are wellacknowledged as a bridge-cell population between innate and adaptive immunity. Moreover, they modulate both tumor cells and anti-tumor immunity [117].The importance of DCs in melanoma immune response and therapy is notorious and intensely exploited [118]. DCs express IL-8Rs and even secrete IL-8 either when are inactive or increased amounts when are activated/matured. The retention of DCs in TME can compromise the induction of anti-tumor immunity. In xenografts of colon carcinoma, DCs were retained within tumors due to IL-8-mediated chemo-attraction, but maintained their abilities to induce T-cell activation. However, recent studies showed that prolonged pre-exposure of DCs to IL-8 diminished their capacity to migrate along IL-8 gradients [119]. *IL-8 and Tregs—*Tregs are a specialized T cell subpopulation that suppress immune response. Tregs maintain homeostasis and self-tolerance and can inhibit T cell proliferation and cytokine production, preventing autoimmunity. They populate lymphoid and non-lymphoid tissues and act during the initiation of inflammatory responses. Although known to express CKRs which attract them to sites where CKs are released, Tregs themselves express and secrete IL-8, which makes them important recruiters of other immune cells [78].

#### 3.3.2. Tumor Cells and Non-Immune Cellular Phenotypes

**Figure 2:**
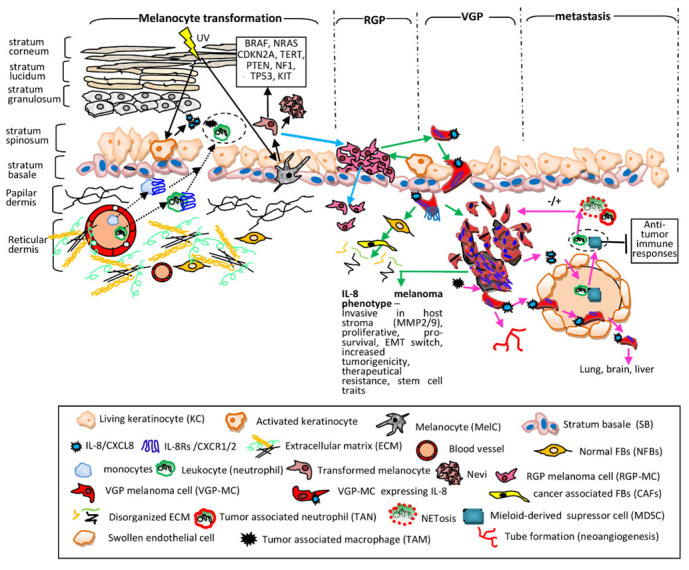
IL-8 contribution to melanoma progression. Melanocyte transformation (black solid arrows)—in KCs the UV light induces secretion of various CKs including IL-8 which trigger extravasation of monocytes and neutrophils into dermis and epidermis (black dotted arrows) creating a pro-inflammatory site (dotted circle) [124,125]. In MelCs UV increases both protective melanin synthesis and ROS generation, destructively affecting MelC genome stability; the resulting transformed cells have mutations (the solid line box), are highly proliferative forming benign tumors (nevi). Neither MelCs nor nevus cells express endogenously IL-8. Radial growth phase (RGP) (blue arrows)—transformed cells proliferate along SB, forming a patch in the skin. The RGP-MCs invade dermis; however, they cannot form a tumor mass. The RGP-stage is generally associated with benignity and RGP-specimens still do not express IL-8. Vertical growth phase (VGP) and IL-8 phenotype (green arrows)—activated KCs together with hypoxia conditions and pro-inflammatory CyKs (IL-1 α, IL-1β, TNF-α) trigger IL-8 expression in RGP-MCs. They switch the molecular repertoire, activate processes which allow them to protrude beyond the SB and acquire IL-8 expression [103]. The MCs inhibitory motility signals are downregulated; MCs become invasive into papillary and reticular dermis, being actively involved in the transformation of NFBs into CAFs; CAFs increase melanoma invasiveness and ECM degradation. MCs expressing IL-8/IL-8Rs grow intensely due to autocrine/paracrine signals, attach to degraded ECM with overexpressed integrins and migrate deep into stromal compartment forming clusters (noduli) and in all these processes IL-8 is actively involved [104,105]. IL-8-VGP melanoma phenotype is correlated with an invasive potential, development of metastasis and poor prognosis. Metastasis (pink arrows)—MCs with IL-8/IL-8Rs encounter circulatory systems. IL-8 mediates paracrine mechanisms between MCs and neutrophils and MDSCs resulting their extravasation into tumor mass (dotted circle) [108]. TANs and NETs have positive or negative consequences on tumor fate [110] whereas MDSCs have intense suppressive anti-tumor immune activities [116]. In addition, TAMs secrete IL-8 and stimulate MCs to release IL-8 and other CyKs [113]. ECs altered because of pro-inflammatory milieu allow extravasation of immune cells, express IL-8Rs and exchange signals with MCs. The IL-8-mediated cross-talk between immune, tumor and ECs assist intravasation of MCs, their maintenance in circulation by adhering to ECs, further extravasation, colonization of secondary places (lung, brain, liver) and neovascularization and tube formation [122,123].

In addition to melanoma and immune cells, the MME contains other host-derived cell types, including keratinocytes, fibroblasts, endothelial cells, and pericytes. In *epidermal keratinocytes (EKCs)*, which control melanocyte proliferation rate, IL-8/IL-8Rs are highly expressed [84] and following UV exposure only CXCR2 became downregulated which reduced the growth of EKCs [120]. In dermis, MCs convert NFBs into *cancer-associated fibroblasts (CAFs)* which contribute to their progression. Unlike NFBs, CAFs are able to elevate the invasiveness of MCs, via mechanisms which involve both IL-6 and IL-8 [121]. *Endothelial cells (ECs)* represent a phenotype strongly involved in mechanisms regulated by IL-8. ECs form the barrier between blood and lymphatic vessels and tissues, control the flow of substances and fluid into and out of a tissue. TCs, including MCs, enter the circulatory system, move along the vessel route and leave them in order to accumulate as secondary tumors in distal tissues. During chronic inflammation, the permeability of endothelium is altered, leading to tissue swelling which in cancer means tumor cell extravasation. ECs are vigorously signaling to white blood cells and to TCs as well. IL-8 is a key mediator involved in activities developed by both ECs and MCs. Human dermal microvascular ECs constitutively express IL-8Rs and IL-8 presence as paracrine or autocrine factor regulates proliferation, survival and secretion of MMP-2/-9 and promotes angiogenetic processes as tube formation and neovascularization [23,122,123]. The step-wise melanoma evolution in relation with IL-8 participation is schematically presented in Figure 2. The melanoma stages, as were described in Section 2, with the principal cellular actors within specific tissular environment including TCs, SCs and cells of the immune system are sequentially discussed from molecular mechanistic point of view.

### 3.4. The Molecular Mechanisms behind IL-8-Mediated Pathways and Processes

The IL-8 bound to IL-8Rs, coupled to Gαβγ activate primary different pathways, which further change the cellular program and eventually the phenotype [126,127]. There is significant evidence for IL-8/IL-8Rs in multiple processes defining melanoma progression, established from the following: (a) in vitro studies on melanoma cell lines, endogenously expressing IL-8/IL8Rs and representative for different melanoma stages, manipulated for the gene expression of *IL-8* [128,129] and *CXCR1*/*2* [130,131,132] or with neutralizing specific anti-IL-8 antibodies [133], and (b) in vivo tumor models constructed with cells expressing high-/low-IL-8 or- IL-8Rs [134,135].

Despite all these well-argued functional assays, there is poor information about the molecularity of IL-8-mediated signaling pathways directly obtained from melanoma cells. There are, however, numerous deciphered mechanisms involving IL-8/IL-8Rs axis in other different neoplasms. As IL-8 and any of these pathways, taken separately, are well-acknowledged actors in melanoma progression, it is thus rational to extrapolate the findings from other cancers to melanoma.

However, we have to keep in mind that each TME, within each neoplasm and moreover within its stages, is different. To all these, the individual variability has to be also taken into account. Therefore, the molecularity behind processes/pathways, although useful to be known, should not be a priori assumed from other neoplasms but rather remains to be individually confirmed and deeply explored in CM.

In addition to these considerations is important to define and understand how the settings of experiments for exploring IL-8 contribution are done. The impact resulted from blocking CXCR1/2, unless demonstrated to be IL-8 specific, may represent cumulated effects on multiple pathways mediated by CKs which are also ligands of these receptors as IL-8 is.

**Figure 3:**
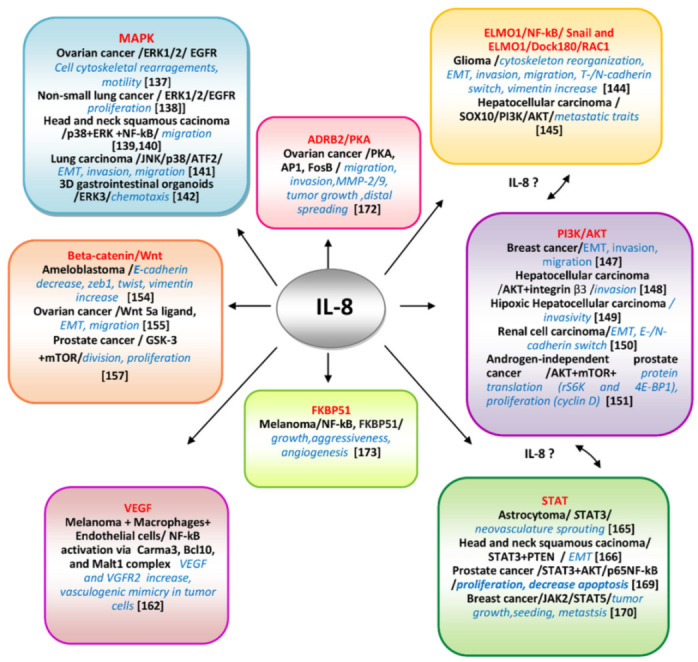
Molecular pathways IL-8-mediated and deciphered in various neoplasms.The main well-documented pathways and key-molecular actors involved in controlling IL-8-mediated processes tumor cell-specific are presented. The communication between different pathways as ELMO1/AKT/STAT possibly mediated by IL-8 are suggested.

The most significant molecular cascades involving IL-8/IL-8Rs axis well-demonstrated in different cancers, including CM, are schematically presented in Figure 3 and will be further briefly discussed.

#### 3.4.1. Mitogen-Activated Protein Kinase (MAPK) Pathways/IL-8

To date, six distinct groups of MAPKs have been characterized in mammals—extracellular signal-regulated kinase (ERK)1/2, ERK3/4, ERK5, ERK7/8, Jun N-terminal kinase (JNK)1/2/3 and the p38 isoformsα/β/γ(ERK6)/δ. They are conserved signaling cascades regulating various cellular processes as proliferation, differentiation, senescence, survival, and transformation. The MAPKs are extensively involved in melanoma progression being supported by the specific mutational repertoire of this neoplasm [136]. The IL-8 involvement has been deciphered in relation with the ERK pathway in SK-OV-3 *ovarian cancer cells* [137], where it has been found that stimulation with IL-8 activated Ras-dependent MAP kinase pathway via phosphorylated EGF receptor (EGFR) associated with adaptor molecules Shc and Gbr2. The IL-8 stimulation induced EGFR transactivation via another non receptor kinase, than c-Src, in these cells. These data demonstrated cross-talks between EGFR and CXCR1/2 activated pathways. The morphological changes in IL-8 SK-Ov3 cells resulted in cytoskeletal rearrangements and impacted cell motility. In *non-small cell lung cancer cell lines* (A549 and NCI-H292) selective inhibitors of MAPK branches demonstrated that IL-8 induces transactivation of EGFR and stimulation of ERK1/2, but not of p38 MAPK, resulting in tumor cell proliferation and involving the expression of a MMP located at PM [138]. In *head and neck squamous cell carcinoma (HNSCC) cells* the p-38 and ERK branches of MAPK were activated compared with normal subjects. In IL-8 stimulated cells increased even more p-38 and ERK phosphorylation levels whereas IL-8 silencing suppressed them. Interestingly, p-c-Jun MAPK branch was the opposite, lower in HNSCC versus normal, reduced and increased after IL-8 stimulation and downregulation, respectively. In IL-8 stimulated cells the expression of MMP-2/MMP-9 and migration was increased and NF-kB activated. All together these data demonstrate that IL-8 is an important factor in the determination of migratory traits via activating the p38 MAPK/ERK-NF-κB pathway and reducing JNK [139]. This selective MAPK pathway activation is important to be further deciphered as it is known that p38 branch can function either as tumor-suppressor kinase, inhibiting RAS-dependent transformation, or as a tumor promoter, and this dual behavior could be p38 isoform-specific [140]. In A549 lung carcinoma cells, IL-8 in conjunction with VEGF from conditioned culture media were demonstrated to be involved in EMT program, including changes in shape from cuboidal to spindle, actin cytoskeleton remodeling, upregulation of vimentin, and downregulation of E-cadherin. The migratory and invasive cellular traits were increased via JNK and p38 phosphorylation and further ATF2 activation [141]. A recent study employing *3D organoids for gastrointestinal tumorigenesis* reveal a dependence of IL-8 expression of ERK3/MAPK6, an atypical component of MAPK family [142]. This is a novel, dual,” both ways” pathway in which a member of MAPKs is controlling IL-8 expression and chemotaxis, and IL-8 in its turn regulates different MAPK-mediated pathways and subsequent events.

#### 3.4.2. Engulfment and Cell Motility Protein 1(ELMO1)/NF-kB/Snail/IL-8 and ELMO1/Dock180/RAC1/IL-8

The multifaceted EMT process in melanoma promotes transition from a proliferative to an invasive state and therapy resistance, being a key driver of metastatic spreading and associated with inflammatory TME [143]. The Rho family of GTPases, including Rac, are modulators of cytoskeleton elements involved in cell migration or invasion events during EMT. The well-known GDP-GTP- switch, a requisite of these classes of modulators is dependent of highly tissue specific guanine-exchange factors (GEFs). A molecular complex, functioning as a GEF for Rac, including engulfment and cell motility 1 (ELMO1) and dedicator of cytokinesis 1 (Dock180) (ELMO1/Dock180) has been identified as a key player in migration and invasion of *glioma cells*. IL-8 regulated the activation of RAC1 which resulted in cytoskeletal reorganization in an ELMO1-dependent manner. IL-8 was also a stabilizator of Snail, a transcription factor involved in mesenchymal transition via ELMO1. Furthermore, the NF-kB pathway, which controls IL-8 expression, was thus demonstrated to be involved in Snail stabilization induced by IL-8. This is an example in which IL-8 is connecting two pathways involved in tumor cell invasion and migration [144]. Worth mentioning that glioma, a brain cancer, is the most related neoplasm with melanoma in terms of aggressiveness and therapeutic resistance. As IL-8 is also sustaining EMT in melanoma it is rational to assume that melanoma and glioma possibly share common IL-8 activating pathways. Moreover, ELMO1 expression was associated with *HCC metastasis* and a poor prognosis and induced EMT via the SOX10/PI3K/Akt axis [145]. It is very likely that ELMO1/IL-8-mediated pathway to establish cross-talks with PI3K/AKT also IL-8-mediated in many neoplasms.

#### 3.4.3. Protein Kinase B (AKT)/IL-8

AKT is a major pathway that promotes survival and growth highly regulated by multiple mechanisms, often involving, as in the case of MAPK pathways, cross-talk with other signaling cascades [146]. In *human breast cancer cells*, IL-8 modulated their invasive and migratory traits, during EMT transition, induced by hormone leptin, via PI3K/AKT signaling pathway [147]. In *hepatocellular carcinoma (HCC) cell lines* IL-8 controlled their invasivity via increased expression of integrin β3 and activation of PI3K/AKT pathway. The IL-8/CXCR1/CXCR2/PI3K/Akt/integrin β3 axis is suggested as a potential treatment target for patients with HCC [148]. Another mechanism described in hypoxic HCC secreting netrin-1, a protein capable to induce EMT in these cells, was involving also PI3K/AKT pathway and the production of multiple inflammatory cytokines, including IL-8, which sustained invasivity [149]. In *renal cell carcinoma (RCC)* the EMT process is highly associated with metastasis. IL-8 is overexpressed in metastatic RCC inducing *E*- to *N*-cadherin switch and enhancing AKT phosphorylation. By inhibiting IL-8 -induced AKT-phosphorylation the EMT of RCC could be aborted [150]. In *androgen-independent prostate cancer (AIPC) cells* recombinant human IL-8 activates PI3K/AKT by increasing both phosphorylation and total AKT. Downstream of AKT, IL-8 activates two mTOR substrates rS6K and 4E-BP1 responsible for promotion of cap dependent translation. When IL-8-mediated pathway was blocked by LY294002 and rapamycin, inhibitors of AKT and mTOR, respectively, cyclin D expression and cell proliferation were severely decreased [151].

#### 3.4.4. Beta-Catenin/Wnt/ IL-8

The Wnt/β-catenin pathway is crucial in numerous pathological states, when its hyper-activation promotes EMT, migration, invasion and proliferation, linked to aggressiveness and worse prognosis [152]. The Wnt/β-catenin signaling is well acknowledged in melanoma pathogenesis [153]. In *ameloblastoma,* a common odontogenic epithelial tumor in the maxillofacial area, in tumor cells IL-8 could promote EMT by decreasing expressions of E-cadherin, increasing phosphorylation of β-catenin and the expressions of EMT-specific transcription factors, twist and zeb1, and of EM -marker, vimentin [154]. In *ovarian cancer cells* the effect of exogenous IL-8 is mediated through upregulation of Wnt5a ligand expression and downstream activation of the Wnt pathway with Met and c-Jun triggering the EMT process and subsequent cell migration [155]. *Glycogen synthase kinase 3* *(GSK-3)* is also integrally tied to pathways of cell proliferation and apoptosis. GSK-3 has been shown to phosphorylate β-catenin thus targeting it for degradation. GSK-3 is therefore a part of the canonical β-catenin/Wnt pathway, which signals the cell to divide and proliferate [156]. In *prostate cancer cells*, IL-8 is able to increase GSK-3β phosphorylation at Ser9 and thus attenuates its activity through an mTOR mediated mechanism [157].

#### 3.4.5. Vascular Endothelial Growth Factor (VEGF)/IL-8

Activation of the Hif pathway produces angiogenic factors within tumor, stromal and inflammatory cells [158]. The reciprocal communication between melanoma and ECs promotes melanoma invasive behavior, sustaining the development of a favorable microenvironment and recruiting vessels [159]. VEGF-A is one of the most proangiogenic factors, with a strong effect on ECs proliferation, survival and migration and on vasculogenic mimicking properties in MCs. Despite the VEGF dependence of activated Hif-pathway there is accumulating evidence suggesting that HIF1-independent mechanisms regulating VEGF expression may also exist [160]. IL-8 has been demonstrated as one of the most pro-angiogenic factor in numerous tumors, including melanoma [161]. The IL- 8 and VEGF secretion is increased in activated macrophages (which secrete also TNF-1α and IL-1α) and in co-cultures of macrophages with MCs. These factors in addition with those produced by ECs are inducing angiogenesis. A study performed on *murine ECs* has explored even in more depth IL-8 contribution and found that overexpression of VEGF and autocrine stimulation of its receptor, VEGFR2, was through activation of NFkB via CBM complex (Carma3, Bcl10, and Malt1) [162]. The activation of VEGFR2, may in turn contribute to the angiogenic response that characterizes the inflammatory processes which are often associated with tumor progression.

#### 3.4.6. Signal Transducer Activator of Transcription (STAT)/IL-8

STAT-3 activity has been associated with increasing the likelihood of melanoma relapse after treatment [163,164]. The IL-8/STAT3 signaling pathway is a regulatory mechanism of angiogenesis in *astrocytoma*, the most aggressive form of glioblastoma, a melanoma-related cancer, controlled by VEGF/Hif-1a axis which ensures neovasculature sprouting [165]. In *head andneck squamous cell carcinoma*, it has been revealed that IL-8-PTEN interaction induces tumor cell progression interfering with STAT-3 pathway, by promoting EMT processes [166]. Importantly, the significance of STAT-3 and PTEN signaling pathways are well acknowledged in melanoma progression [167,168]. However, so far, they have not been connected to IL-8. As PTEN is also a modulator of AKT pathway a cross-talk between STAT3 and AKT mediated by IL-8 could be expected. In *prostate cancer cells* the overexpression of IL-8 induced proliferation, invasion and decreased apoptosis via STAT-3/AKT/NF-kB by increasing phosphorylation of STAT3, AKT and NF-kBp65 [169]. Another pathway involving IL-8 was related to PI3K/mTOR inhibition which has activated JAK2/STAT5 and IL-8 secretion in *breast cancer cell lines and tumors*. The data of this study demonstrated that inhibition of the JAK2/STAT5/IL-8 which occurred after PI3K/mTOR blockade decreased tumor growth and lung metastasis. This approach targeted in particular a metastatic subpopulation of cancer cells responsive to IL-8 stimulation via CXCR1 [170].

#### 3.4.7. ADRB2/PKA/IL-8

Adrenergic signaling plays a fundamental role in chronic stress-induced tumor progression and metastasis [171]. Under stress the sympathetic nervous system (SNS) is releasing neurotransmitters, such as catecholamines, which act on Beta-adrenergic receptors (ADRB2) activating different cellular pathways. In *ovarian carcinoma cells (OCs),* SKOV3ip1 and HeyA8, has been demonstrated that IL-8 production was mediated by adrenergic ADRB2/PKA signaling through transcription factor AP-1. The *IL-8* overexpression, controlled by FosB, led to increased invasion and migration of tumor cells whereas no effect on tumor cell proliferation or cell cycle were detected. This in vitro hypothesis of stress induced OC metastasis via an IL-8 mediated mechanism was confirmed by in vivo model of injection SKOV3ip cells into the mice ovaries and exposure to daily restraint stress. The results showed significant growth of tumor nodule counts and distant metastatic spread compared with no stressed controls. Downregulation of *IL-8* expression levels in stressed mice resulted in lower levels of MMP-2 and MMP-9 in the tumor microenvironment and abolished the effect of stress on tumor metastasis [172].

#### 3.4.8. FKBinding Protein 51(FKBP51)/IL-8

A molecular pathway regulated by IL-8 and involving FKBP51 has been deciphered directly in melanoma cells [173]. In addition of being a member of highly conserved family of immunophilins, FKBP51 is also a co-chaperone associated with Hsp90 and a key player in stress-related disorders, obesity and chronic pain [174]. FKBP51 is over expressed in melanomas associated with survival, chemo-resistance [175,176], stemless and metastatic potential [177]. The FKBP51 analysis in melanoma demonstrated that: FKBP51 regulates IL-8 through the activation of NF-kB, and in turn, IL-8 mediates in part FKBP51-regulated melanoma growth, aggressiveness and the effect of FKBP51 on angiogenesis in melanoma. This is an example of cross-talk in melanoma molecularity. Our group has also identified such a reciprocal modulating mechanism in melanoma cells between caveolin-1, a major regulator of signaling platforms and dopachrometautomerase, a melanoma antigen with multiple functions, including melanoma resistance to therapeutical and environmental stress [178]. Importantly, this IL-8 mediated pathway has not been described, so far, to our knowledge, in other neoplasms.

All this information demonstrates one more time that behind cancer ubiquitous pathways and mechanisms there is a unique molecularity, cancer-type specific, which is finely tuning each event.

### 3.5. IL-8-Mediated Pathways and Processes in the Prognostic and Therapy of CM

As CM still represents a life threat neoplasm the treatment strategies of melanoma are continuously evolving based on the most recently unveiled mechanisms, key players and biomarkers [179]. IL-8 is the prerogative of pathological states and is practically undetectable in normal tissues. The pro-tumoral actions of IL-8 exerted on MCs and immune cells associated with host response, as well as on MME, makes it an interesting possible biomarker and therapeutic target.

#### 3.5.1. IL-8 Serum Levels

Lactate-dehydrogenase (LDH) is the only serological biomarker included by AJCC in TNM melanoma staging system and it prognosticates the patients with metastatic stage [180]. Serological analysis of CyKs, CKs, and GFs from malignant melanoma patients indicated a direct association between the levels of IL-6, IL-8, IL-13, VEGF and the values of the tumor thickness [181]. Early studies reported IL-8 presence in the serum of patients with different cancers, including melanoma [182], and IL-8 has been used as prognostic biomarker for different tumors [183,184]. The quantified serum levels of vitamin D3, IL-8 and LDH showed lower and higher concentrations of vitamin D and IL-8/LDH, respectively, in melanoma patients than in healthy donors. In correlation with vitamin D3, IL-8 could improve prognostic by identification of high risk subgroups [185]. A study with 125 melanoma patients correlated the serum levels of IL-8, basic Fibroblast Growth Factor (bFGF) and VEGF with stage of disease and tumor burden. IL-8, bFGF and VEGF were considerably increased in the metastatic stage of disease compared to primary melanoma, being associated with poor overall survival [186]. In addition to molecular biomarkers IL-8 expression was correlated with presence and number of certain immune cell phenotypes. For example, the immunosuppressive MDSCs, that express IL-8Rs and are chemoattracted to IL-8. The elevated serum levels of IL-8 in metastatic melanoma patients (stage IV) have been associated with a higher frequency of MDSCs in the circulation [116]. The increased serum IL-8 is also directly linked to levels of circulating neutrophils and monocytes [187]. IL-8 has been found an accurate molecular tool to monitor the effects of different anti-cancer treatments, in different neoplasms, especially in situations when imagistic cannot provide relevant information. For melanoma, the data showed the followings: (a) in culture supernatants, IL-8 levels were quantitatively correlated with MC numbers and mRNA for IL-8 was increased in hypoxic state; (b) inxenografts IL-8 serum levels were precisely correlated with tumor volume and upon excision its level dropped; (c) the patient serum levels were correlated with tumor size and stage; (d) IL-8 levels can be used to monitor tumor stage following treatments with different combinatorials which do not target its expression directly. The study concludes that “IL8 showed a better correlation with tumor burden in the progression of the disease in patients with melanoma than LDH, the classical marker in this disease” [188].

#### 3.5.2. IL-8 and CM Therapy

There are multiple and complex anti-tumor therapeutic strategies including CyKs. In combinatorials, CyKs are directly associated with chemo-/radio-therapies, or with other immuno- or targeted therapies. Alternatively, the CyKs are produced at tumor site, as is type I IFN or IFN-γ, by stimulating immune cells with specific up-regulators as toll-like receptor agonists [189]. For CM, the FDA approved in trials immunotherapies include so far: (a) interferon (IFN) α-2b [190], (b) polyethylene glycol(PEG)interferon α 2b (PEG-IFN), a combination of IFN α -2b with PEG ) [191] and (c) IL-2 [192].

We witness comprehensive reviews which provide data about different IL-8-targeted cancer therapies, including melanoma and urge the need for even more novel combinatorial ones [193]. The most significant preclinical and clinical studies on cell cultures, animal models or patients about CM and other neoplasms treatments involving IL-8 and their potential development are collated into Table 2 and further presented.

##### Blocking IL-8 Expression

In preclinical studies, the monoclonal antibody (mAb) directed against human IL-8, ABX-IL8 (Abgenix, Inc., Fremont, CA) is able to neutralize the IL-8 secreted by MCs. The results were: inhibition of MC invasion through basement membranes, and MMP-2 secretion as well as decreased vascularization. Overall, ABX-IL8 suppressed the tumorigenicity and metastatic potential of metastatic human melanoma A375SM and TXM-13 cells in vivo, on animal models [133]. In a more advanced study the effect of ABX-IL-8 in combination with an antibody against a melanoma cell adhesion protein, MUC18, and dacarbazine, the gold standard anti-melanoma drug, is suggested as a novel treatment that could overcome metastatic melanoma resistance to chemotherapy and improve survival of patients [194]. Alternatively, IL-8 downregulation by siRNA has been exploited to potentiate response to docetaxel in ovarian tumor xenografts [195]. This suggests that depleting IL-8 by siRNA, instead of using ABX-IL8, could potentiate dacarbazine treatment in melanoma as well.

##### Blocking IL-8Rs

An efficient approach of IL-8 blockade is to target directly IL-8Rs. Studies with neutralizing antibodies to either CXCR1 and CXCR2 showed inhibition of MC proliferation and invasive potential whereas in athymic nude mice oral administration of CXCR1/2 antagonists, inhibited the growth and angiogenesis of implanted human melanoma tumors [196]. Reparixin is a CXCR1/2 inhibitor currently under treatment of metastatic triple negative breast cancer (Phase 2) [197]. Ladarixin (LDX) is a small molecule with high affinity for CXCR2. LDX decreased melanoma progression by multiple effects including inhibition of tumor survival pathways, impacted immune cell recruitment and abrogated tumor cell renewal and angiogenesis [198]. We reiterate that blockades of CXCR1/2 may include contribution of other CKs which share these receptors with IL-8.

##### Combinatorials with Impact on IL-8/IL-8Rs Axis

*BRAF and MEK inhibitors*—the drugs vemurafenib (PLX4032; Zelboraf), a V600EBRAF inhibitor, and trametinib (GSK1120212; Mekinist), a MEK1/2 inhibitor, approved by FDA for the treatment of patients with advanced melanoma harboring mutation in BRAF (V600E) have been tested on six melanoma cell subpopulations. The most severe impact of both vemurafenib and trametinib among investigated genes was on *IL-8* expression [199]. Importantly, despite these suppressive effects exerted on the pro-tumor mediator IL-8, an unwanted consequence of this targeted drug-induced therapy was, in turn, the expression of CD271, a marker of melanoma stem-like cells. This example demonstrates, one more time, that versatile TCs upregulate additional mechanisms when are attacked. This report advocates for supplementation of the present combinatorial with potent drugs directed to cancer-stem cells.

*Anti-Program Death (PD) Pathway and anti-CTLA-4 mAbs*—the PD pathway refers to one of the check points which drive melanoma progression. PD-L1, frequently expressed within MME, including by cancer cells, binds to PD-1 on T cells and its binding triggers subsequent inhibitory signals which block T-cell-killing activity. Targeting PD1/PDL1 axis proved useful in inducing the stimulation of immune cells to attack the tumors [200]. Three mAbs nivolumab (anti-PD-1) pembrolizumab (anti-PD-1) and atezolizumab (anti-PD-L1) have been approved for the treatment of different tumor types including melanoma [201,202,203]. Cytotoxic T Lymphocyte antigen -4 (CTLA-4) is a negative key regulator of T cell activation. The aberant upregulation of CTLA-4 hijacks the T cell signaling and compromise T-cell responses. It was demonstrated that CTLA-4 blockade could attenuate the growth of several implanted murine tumors. Ipilimumab (MDX-010, Yervoy; Bristol-Myers Squibb), a fully human mAb against CTLA-4, was approved by FDA for thetreatment of metastatic melanoma [204] and in patients with melanoma and preexisting auto immune disorders [205]. In a combinatorial study, anti-PD-1 mAbs, used either as single-agent or in combination with anti-CTLA-4 mAbs have been tested on patients with melanoma and non-small cell lung cancer (NSCLC) and IL-8 levels were monitored [206].

The results showed that serum IL-8 levels correlate with tumor burden changes in metastatic melanoma following treatments with anti-PD-1 mAbs plus anti-CTLA-4 mAbs. Moreover, IL-8 levels were able to discriminate between patients with real and pseudo-progression.

##### Combinatorials with Impact on IL-8-Mediated Tumor Immune Cellular Milieu–MDSCs

The outcome for melanoma patients is highly correlated with MDSC and subsequently with the regulatory mechanisms which control their fate, including secreted CyKs. Patients with high expressions of IL-8/IL-6 in tumors and plasma and frequency of circulating MDSCs have worse median survival than patients with low levels of these parameters. Whereas the IL-6 impact is to sustain expansion of MDSCs, the IL-8 contribution is to attract these tumor suppressor cells at MME [116]. The consequence of MDSC accumulation is also the reduced numbers of DCs. The conclusion of a comprehensive review about the roles of MDSCs in melanoma progression is that “a key prerequisite for an effective melanoma immunotherapy should involve the neutralization of MDSC-mediated immunosuppressive microenvironment typical for melanoma before applying any further immunologic treatments” [207]. Taking into account the role of IL-8 in the fate of MDSCs, it is rational to add that an anti-melanoma therapy including an anti-IL-8 component could aid the neutralization of MDSCs. *DCs*—The effects of IL-8 on DCs were explored with IL-8 neutralizing mAb and in immunodeficient mice xenografts with colon carcinomas having downregulated *IL-8* gene expression. DCs injected into HT29 or CaCo2 xenograft tumors were retained intratumorally in an IL-8-dependent classical chemotaxis fashion [119].

Functional IL-8 did not modify the ability of DCs to stimulate T cells. However, interestingly, pre-exposure of DCs to IL-8 desensitizes such cells for IL-8-mediated in vitro or in vivo chemo attraction. DCs were unable to follow IL-8 chemotactic gradients towards malignant or inflamed tissue. The authors suggest that lower IL-8 levels with either chemical compounds or anti-IL-8 antibodies could aid benefits to therapies with DCs. Unexpectedly, a very recent study (in vitro and in vivo) on colorectal cancer (CRC) demonstrated that activation or recruitment of DCs was impeded by blocking CXCL8-CXCR2 axis with CXCR2 antagonists. The authors suggest that IL-8/CXCR2 axis is “a favorable factor rather than a target for critical antitumor effects on CRC” [208] which is somehow the opposite of what was so far known about IL-8. *PMNs and TANs*—Co-injection of melanoma cells with human PMNs stimulated with LPS, which turned on IL-8 production, resulted in the quick formation of tumor cell-PMN heterotypic aggregates along the endothelial layer by both mechanical trapping and neutrophil endothelial adhesions [209]. Reducing IL-8 expression with siRNA decreased the number of MCs bound to endothelium and abrogated their extravasation and metastasis [210]. The phase I clinical trial (Study NCT02001974) of patients with HER-2 negative metastatic breast cancer Reparixin, an orally available inhibitor of IL-8Rs was successful in combination with paclitaxel [211]. Both Paclitaxel and blockade of IL-8Rs were separately used in melanoma therapy, so such a combinatorial would be expected successful in melanoma too. Additional chemotaxis inhibitors, as those targeting CXCR2, an important marker for neutrophil migration from bone marrow into site of inflammation, in combinations withother inhibitors of CKs/CKRs (e.g., CXCL12/CXCR4 or CCR5-maraviroc) are under investigation to hinder the recruitment of neutrophils to the TME [212,213]. *TAMs*—Numerous studies showed that TAMs which favor tumor progression resemble to macrophage M2 phenotypes [214]. In order to protect normal macrophages and avoid liver cell toxicity, the reprogramming of M2 to pro-inflammatory M1 phenotypes would represent a therapeutical option. The utilization of a mAb anti-CD40, CP-870893, targets the non-ligand binding site of CD40, enhance the secretion of IL-12, IL-23, and IL-8 and in combination with gemcitabine, is associated with antitumor activity in pancreatic ductal carcinoma patients [215]. More combinatorials are used to induce pro-inflammatory gene expression in TAMs. An inhibitor of PI3Kg in combination with nivolumab is in Phase 1b clinical trial for solid tumors [216]. Ibrutinib, with its inhibition on targets downstream of PI3Kg, can induce pro-inflammatory polarization of macrophages as well as CD8^+^ T cells infiltration and in combination with chemotherapeutic compounds are in clinical trials for treatment of pancreatic adenocarcinoma relapsed or refractory solid tumors. Although not directly related to IL-8-mediated pathways, TAMs in melanoma can be targeted by trabectedin, an antineoplastic drug, which induces death of monocytes/macrophages via a TNF-related apoptosis-inducing ligand (TRAIL)-mediated mechanism, restricting thus melanoma growth and metastasis via reducing the number of TAMs in the tumor microenvironment [217]. Another study suggests that specific depletion of the CD163^+^ macrophages can trigger tumor shrinkage in a melanoma model by increasing infiltration of effector T cells and concomitant recruitment of pro-inflammatory TAMs [218].

**Table 2:** Strategies of targeting IL-8/IL-8Rs in therapies of CM and other neoplasms.

Target	Therapeutic Strategy	Impact	Experimental Approach	References
**IL-8**	mAb anti h-IL-8 (ABX-IL8)	Neutralize secreted IL-8; inhibit invasion, MMP-2 secretion, decrease vascularization	Human melanoma and animal models	[133]
	IL-8 neutralizing Abs *IL-8* downregulated	Disorients DC migration, without impairing T-cell stimulation	Colon cell carcinoma tumors	[119]
**IL-8Rs (CXCR1/2)**	Inhibitors			
	Low-molecular-weight antagonists, modified chemokines, antibodies directed against receptors	Inhibit tumor growth and angiogenesis	Human melanoma tumors in athymic nude mice	[196]
	Antagonists to CXCR2	Promotes tumor progression in vivo by impeding DC activation or recruitment	Colorectal cancer subtype	[208]
	Ladarixin	Abrogates tumor cell motility, self-renewal, intratumor de novo-angiogenesis; induces apoptosis, polarizes M1 TAMs	Melanoma cells, xenografts and tumors	[198]
	Reparixin	Reducing cancer stem cells by targeting their CXCR1	Breast cancer clinical trial phase 3	[197]
**COMBINATORIALS** **IL-8/IL-8Rs agonists, mAbs, gene downregulators, tumor** **specific pathways/key molecules, chemotherapeuticals,** **immune cell modulators**	Braf inhibitor/vemurafenib; MEK1/2 inhibitor/ trametinib	Decrease *IL-8* and suppress tumor evolution	Melanoma cell subpopulations	[199]
	mAb (ABX-IL8) + MUC18 + DITC	Overcome resistance to chemotherapy and improve survival of patients	Metastatic melanoma	[194]
	Si-IL-8 treatments + docetaxel	Downregulate *IL-8* and potentiate chemotherapeutic agents	Ovarian tumor xenografts	[195]
	mAbs anti-PD1/PD-L1- (nivolumab, pembrolizumab, atezolizumab) + mAbs antiCTLA-(Ipilimumab)	Tumor burden changes	Patients with melanoma and NSCLC	[206]
	Reparixin + paclitaxel	Increases tumor sensibility to chemotherapy	HER-2 negative metastatic breast cancer	[211]
	Inhibitors of CXCR1/2 + CXCL12/CXCR4 or CCR5	Hinder recruitment of neutrophils in tumor microenvironment	Metastatic colorectal cancer	[213]
	mAb anti-CD40 + gemcitabine; PI3Kg inhibitors+ nivolumab	Increases pro-inflammatory gene expression in TAMs, reprogramming of M2 to pro-inflammatory M1 phenotypes, anti-tumor activity	Pancreatic ductal carcinoma	[215]
	Synergistic PI3K/mTOR and JAK2 /STAT5 inhibition	Reduced cancer cell number and tumor growth, decreased tumor seeding, metastasis, increased overall survival of the animals.	Breast cancer	[170]

Abreviations: mAb—monoclonal antibody; BM—basement membrane; DITC—dacarbazine; NSCLC—non-small cell lung cancer; MDSCs—myeloid-derived suppressor cells; DCs—dendritic cells; PMNs—polymorphonuclear neutrophils; TANs—tumor associated neutrophils.

## 4. Conclusions and Perspectives

Within almost thirty-five years of its discovery, the scientific portrait of IL-8 has been significantly changed. Numerous IL-8 characteristics are now well-acknowledged whereas even many more still await further clarifications. Undoubtedly, IL-8 is emblematic for pathological states. Explored initially in inflammatory diseases, IL-8 investigation has rapidly extended in tumor area, since both pathologies are regulated by many common factors. In melanoma is widely accepted that IL-8 and IL-8Rs are well expressed by invasive, anti-apoptotic tumor cells. Moreover, various other cells from MME are executing IL-8/IL-8Rs-mediated programs which are finalized with accumulation of immune infiltrates at tumor site, melanoma cell intravasation in vessel fluids as well as their extravasation and tumor noduli formation at secondary sites. The IL-8/IL-8Rs axis acts also on most immune cell phenotypes from which some have either pro- or anti-tumor activities, or both. In addition, the stromal cell phenotypes present in MME are reprogrammed by IL-8-mediated mechanisms and become extremely active in tumor fate. They are suppliers of different factors which impact both tumor cells and immune cell gallery.

Following our broad journey through IL-8 role in melanoma versus other neoplasms, at first glance it can be concluded that IL-8/IL-8Rs axis operates similarly in all cancer types. However, the molecularity behind these events may reveal tumor-specific actors, which so far have not been sufficiently investigated. As melanoma is an extremely heterogenic tumor, unique molecular regulators and pathways which operate in different stages and categories of individuals are expected. Importantly, rather than being analyzed as an individual CK, IL-8 action has to be integrated into general complex puzzle of CyKs which control all the paths along melanoma progression. The emerging reports, apparently controversial at this moment, about IL-8 and TANs, or IL-8 and TAMs should be more attentively deciphered and the relation with DCs as well. They may represent possibilities to turn IL-8, a traditional pro-tumor CK into a pro-patient one. More genomic-wide transcriptomic analysis, justifiably awaited, will offer clarifications about gene clusters regulated by IL-8/IL-8R axis in different melanoma stages and subtypes.

The novel melanoma prognostic and therapeutical strategies based on IL-8 have to include updated, specific melanoma molecular regulators in combination with the already validated chemo-/immuno-therapies. An integrated view is expected to emerge from biostatistics and bioinformatics as the advanced management of melanoma will definitely rely on even more multi-parameter strategies than the current ones.

## Data Availability

Not applicable.
